# Improving Gifted Talent Development Can Help Solve Multiple Consequential Real-World Problems

**DOI:** 10.3390/jintelligence9020031

**Published:** 2021-06-13

**Authors:** Jonathan Wai, Benjamin J. Lovett

**Affiliations:** 1 Department of Education Reform, University of Arkansas, Fayetteville, AR 72701, USA; 2 Department of Psychology, University of Arkansas, Fayetteville, AR 72701, USA; 3 School Psychology Program, Teachers College, Columbia University, New York, NY 10027, USA; BL2799@tc.columbia.edu

**Keywords:** innovation, talent selection and development, gifted education, social returns, cognitive aptitudes and creativity

## Abstract

Fully developing the talents of all students is a fundamental goal for personal well-being and development and ultimately for global societal innovation and flourishing. However, in this paper we focus on what we believe is an often neglected and underdeveloped population, that of the gifted. We draw from the cognitive aptitude and gifted education research literatures to make the case that solutions to consequential real-world problems can be greatly enhanced by more fully developing the talents of the intellectually gifted population, which we operationalize in this paper as roughly the top 5% of cognitive talent. Should well-supported high achievers choose to solve them, these problems span health, science, economic growth, and areas unforeseen. We draw from longitudinal research on intellectually precocious students and retrospective research on leaders and innovators in society, showing that mathematical, verbal, and spatial aptitudes are linked to societal innovation. We then discuss two remaining fundamental challenges: the identification of disadvantaged and marginalized groups of students who have traditionally been neglected in selection for gifted programming suited to their current developmental needs, and the building of skills beyond academic ones, specifically in the related areas of open-minded thinking and intellectual humility.

## 1. Introduction and Roadmap

Solving consequential real-world problems would ultimately best be served by fully developing the multitude of talents of *all* individuals in society. Thus, we should without question help all students, through education and other means, to develop to their full potential. In this paper, we focus on what we believe to be an often neglected and underdeveloped population that very likely could contribute greatly to solving real-world problems to a much larger degree than they currently do (Benbow and Stanley 1996; Gardner 1961). This is the intellectually gifted population, which we operationalize as roughly the top 5% of achievers globally. Systemic and structural barriers reducing the likelihood that many talented but disadvantaged students from low-income and minority backgrounds can ultimately develop their talents and eventual expertise to the fullest is a crucial ongoing challenge (Peters 2021). When many children come from poverty, they will not only fail to be recognized as gifted, they might not even develop to be gifted (e.g., Hair et al. 2015). This is true for countries around the world where lack of opportunities and numerous headwinds (Stevens 2020; Wai and Worrell 2020) face talented but disadvantaged students (in particular compared to their advantaged counterparts). These inequalities in opportunities and challenges may have been even further exacerbated by the COVID-19 pandemic and related learning losses globally (e.g., Hanushek and Woessmann 2020), adding up to a cumulative disadvantage over time. Of course, whether talented students choose to solve consequential real-world problems or do whatever else they want with their lives is entirely up to them. Our hope is that at least for some, choices to fulfill one’s potential might also be consonant with an interest in contributing to the broader improvement of society, and it is in that hope that we write this article.

This special issue call for papers asked contributors to take one consequential real-world problem and discuss what we know about cognitive abilities that could help us to solve the problem. We reframe this question slightly to consider two areas of research informed by cognitive abilities that can help us solve *multiple* consequential real-world problems. First, we review the literature making the case that fully developed gifted students in fact already do very likely solve multiple consequential real-world problems but do so broadly very likely based on their personal interests, life circumstances, and educational and developmental trajectories in different areas of achievement and expertise. We further make the case that more fully developing the talents of gifted students or the top 5% of achievers will likely enhance the likelihood of solving real-world problems in the future. Another core problem is identifying and developing the talents of talented but disadvantaged students, especially underrepresented minorities, to ensure personal development and flourishing but also to broaden the talent pool to solve problems from a broader array of perspectives and personal talents. Broadly, we begin our article describing how developed cognitive aptitudes are important to solving real-world problems, introduce our theoretical and empirical perspective that frames the remainder of the article, and discuss issues in regard to the support and development of gifted students, and really all students, on multiple dimensions.

## 2. Talent Development and Innovation

In 1957 a group of scientists from the California Institute of Technology, after multiple discussions with industrialists and other leaders, published their forecasts for the most important problems facing humanity for the next 100 years. The authors (Brown et al. 1957, p. 152) concluded that “The problems which we face in the years ahead are indeed both numerous and grave, but, theoretically at least, it seems likely that they can be solved by the proper application of our intelligence.” There are many strategies for applying cognitive aptitudes to real-world problems. In this article, we emphasize the importance of investing in all students, with a focus on strategies involving investment in gifted students, in particular those from disadvantaged backgrounds. Cole (2016, p. 23) described the “law of the 5 percent” as the idea that in nearly every field, the top 5% of that field will be responsible for the vast majority of innovation. We introduce the idea of investing in developing more students to be among what is currently the top 5 percent of achievers, then fully developing that broader group of achievers, who we argue have been, are, and will be largely responsible for innovation across multiple fields of intellectual and creative endeavor in the future.

Talented individuals innovate in a variety of ways that can benefit society, and are very likely to rise to positions of influence to be able to implement those innovations (Lubinski and Benbow 2020; Wai and Worrell 2016; Wai 2013). Innovations come from individuals throughout the cognitive aptitude range, and many high achieving students do not choose to solve consequential real-world problems. This suggests we should invest in developing the talents of all students, including gifted students, because as cognitive aptitudes rise, so does the *likelihood* of innovation.

Certainly, the idea of ensuring talent development is not new (Gardner 1961), and is truly a global consequential real-world problem. As researchers who work and live in the US, we are biased towards our local perspective, but also see how in many less developed nations the lack of talent development may even be more severe given greater structural and other barriers such as poverty and lack of opportunity. The US already has a number of programs for the purpose of talent development, both at the level of individual schools and at a national level. However, the availability of talent development programs varies widely. Many schools lack such programming, and the national programs have limited capacity and are often quite expensive. In a broad sense, talent development is the essence of all education (e.g., Subotnik et al. 2011). However, there remain many students with high potential who simply were not born into circumstances with sufficient opportunities, and whose talent is often overlooked and underdeveloped (Hair et al. 2015; Peters 2021).

In the US, this is at least in part because there remains very little federal support for gifted education (Benbow and Stanley 1996), or even any federal requirements to provide such services. Instead, the decision is up to states and school districts, and the availability of services varies widely across these settings. In many school districts, no formal gifted supports are available at all. Even when some supports are present, they rarely include all the students who should be eligible. Often, those left behind are talented students from low-income and historically marginalized backgrounds (Wai and Worrell 2016) and students with overlooked spatial talents (Lakin and Wai 2020; Wai and Lakin 2020). Some scholars argue that COVID-19 learning losses could add up to trillions (Azevedo et al. 2020). Other scholars argue that the long-run economic impact of this loss is the same as one-third of a year of schooling which translates to a gross domestic product (GDP) loss of 1.5% on average for the remainder of the century (Hanushek and Woessmann 2020; Schleicher 2020). In this context, it is crucial to ensure that talented but disadvantaged students do not get left behind.

To be clear, we should invest in all students throughout the full spectrum to develop and help them use that cognitive potential to the very best of their capacity. However, major societal problems are, again, more likely to be solved by those with the greatest developed talents, and when such problems are solved, everyone can benefit. Gifted education should therefore not be viewed as an individual reward to students for having high ability, but perhaps in part as a societal investment with a high likelihood of good returns. Even merely more optimal matching of high-aptitude individuals to jobs and settings that require the solution of complex problems is associated with more economic growth across countries (Strenze 2013). If we go beyond this matching process to actually fully develop the gifts of those with the highest developed potential, this might even lead to even greater gains. Of course, whether individual students choose to pursue certain life courses is ultimately up to them, whether that means taking advantage of opportunities that are available, finding a domain that suits their interests and aptitudes, or sustaining the years of motivation and hard work often required to attain expertise in a given domain.

## 3. Cognitive Aptitudes and Giftedness: Definitions

Though there are numerous verbal operationalizations of what being gifted means (e.g., for a review see Subotnik et al. 2011), we focus on aspects of giftedness that are measurable through cognitive tests as one indicator of giftedness. More specifically, we focus on a version of the hierarchical model of abilities (Carroll 1993) known as the Radex model (Lubinski 2004), which includes general reasoning at the apex and the specific aptitudes of mathematical, verbal, and spatial. This well-established structure, at least in our view, should at least be considered part of a measurable and consistent definition of intellectual giftedness (Coleman and Cureton 1954; Detterman 1993; Thompson and Oehlert 2010; Kelley 1927). We also view all abilities as developed and that cognitive aptitudes are *current* developed capacities that an individual brings to learning or problem-solving environments at a given time (Lohman 2005; Snow 1996). All aptitudes or abilities are thus developed and malleable (Subotnik et al. 2011; Uttal et al. 2013), and they are both important to learning and problem-solving environments such as schooling, but also an important *product* of schooling (Ceci 1991; Lohman 1993; Ritchie and Tucker-Drob 2018).

## 4. High Developed Aptitudes Can Often Lead to Greater Innovation

Even just a small number of academically gifted and talented scientists can improve our lives in the most remarkable of ways. Pinker (2018) summarized findings from scienceheroes.com, which lists roughly 100 individuals with remarkable achievements who have made life-saving discoveries. Based on this data, Pinker (2018) argues that over 5.5 billion lives have been saved by a small cohort of 100 or so individual scientists. This includes the discovery of the chlorination of water, smallpox eradication strategy, measles vaccine, penicillin, oral rehydration therapy, among numerous other examples. The scientists who developed the Pfizer/BioNTech COVID-19 vaccine, Katalin Kariko, Ugur Sahin, Albert Bourla, and Ozlem Tureci are contemporary examples (Gelles 2020).

Rindermann and Thompson (2011) illustrated that the cognitive 5% of a nation’s population disproportionately influenced innovation and GDP of that nation. Longitudinal studies focused on the gifted population also illustrate that fully developed gifted students can earn doctorates, publications, patents, and even university tenure at the rate of two to eight times that of the general population (Lubinski and Benbow 2006, 2020; Park et al. 2007). Findings within the top 1% of aptitudes are replicated in both nonrandom (Lubinski and Benbow 2020) and random gifted samples (Wai 2014). There does not appear to be a threshold beyond which more aptitude no longer matters for a wide range of life outcomes both within gifted samples (Lubinski and Benbow 2020) and also across multiple population-representative samples in the US and UK (Brown et al. 2021). Even when drawing from a large sample of US leaders across a variety of domains such as business, the media, politics, law, and those with enormous wealth, when retrospectively profiling where these leaders attended higher education, roughly half attended educational institutions that largely selected for the top 1% of aptitude on standardized admissions tests (Wai 2013).

## 5. Improving Gifted Talent Development has the Potential to Enhance a Wide Range of Innovations and Social Returns

Innovation can be considered to be largely about creating something truly new and useful. Flexner and Dijkgraaf (2017), as well as Braben (1994, 2020), argued that a key for intellectual advancement is to encourage brilliant and unique minds to pursue whatever interests them—or even what goes against the current popular research topics—and to choose questions that do not necessarily have immediate application. Differential psychology (Revelle et al. 2011) shows us that people have varying interests (Su 2020), and this is true within the gifted population as well (e.g., Lubinski and Benbow 2006, 2020; Wai 2013), suggesting that different interests may be linked to wide ranging areas of innovation. Studies on cohorts of intellectually talented youths in the top 1%, top 0.5% and top 0.01% of aptitude show that as the average talent of the gifted cohort rises, so does the accomplishments of that group (Lubinski and Benbow 2006, 2020). Crucially, the range of innovation of these talented youths is spread across a wide array of domains, from science, technology, engineering, and mathematics (STEM) fields to the humanities, heads of business, partners in law firms, and publication of novels. Coupled with the findings that the top 1% of academically gifted individuals who attended highly selective institutions make up roughly half of various US leaders of society (Wai 2013), this suggests that high ability individuals innovate across a wide range of areas, perhaps based in part on aptitudes (both level and pattern), interests, personality, motivation, and of course their access to appropriate educational or other stimulating opportunities (Wai et al. 2010).

Jones (2016) argues that investment in developing the talents of all individuals in a nation could have positive spillovers in the form of increased patience, cooperation, and being more knowledgeable and informed. Therefore, investments in nutrition and education may have the potential to improve a wide range of outcomes. Jones and Summers (2020, p. 34) assessed the social returns to innovation, concluding that “innovation investments can credibly raise economic growth rates and extend lives, paying for their costs many times over. And because the social returns exceed the private returns, public policy has a central role, and opportunity, in unleashing these gains.” Linking these economic estimates of spillover effects of broad human capital investment to Heckman (2000) payoff curves and broader literature (e.g., Lubinski and Benbow 2006, 2020) showing that fully developed talented individuals may contribute a great deal to innovation in society suggests that investing in the gifted—in particular the less advantaged—has the potential to enhance real-world problem solving and improve the rate of social returns. Admittedly: to ensure that everyone benefits requires social policies that go far beyond gifted education and talent development to address a wide range of inequalities (e.g., Blanchard and Rodrik 2021). Moreover, even when everyone benefits from innovation and advancement, some groups may benefit more than others, widening gaps that already exist (Ceci and Papierno 2005). We do not want to minimize these complex issues; our point is simply that solving major real-world problems has the potential to benefit everyone. Of course, giftedness can be put to bad uses as well as good ones; in Section 8 below, we discuss how to promote the latter applications of giftedness.

## 6. Companies Seek Talented People, Who Can Come from Anywhere

Investing in gifted children in the early years, especially those from disadvantaged backgrounds, can simultaneously help improve innovation and equity. However, in the US at least, gifted education appears to be a low priority in kindergarten through 12th grade (K-12) education. This is in contrast to the broader talent selection and development priority of companies worldwide—including in the US—who are desperately seeking talented individuals from around the world to improve innovation and revenue generation (Roose 2014). For example, global talent searches in the form of high-end programming competitions—Google’s CodeJam, Facebook’s Kaggle Recruit, or Microsoft’s Code4Bill talent search in India—are useful, cost-effective screening tools for top tech companies to get the variety of talented people they need. Google’s CodeJam winner in 2012 described the content of the competition as “more like mathematical work or solving logic puzzles,” so something very much akin to a high-level cognitive aptitude test (Chabris and Wai 2014). Similarly, the Thiel fellows program gives $100,000 and access to a network of contacts to those who want to build things and may not need to go through the traditional sequence of schooling such as attending college (https://thielfellowship.org/, accessed on 10 June 2021). Recently, Eric and Wendy Schmidt launched the Rise program, which seeks to uncover talented youths from around the world and provide them with resources for life (Mehta 2020).

Companies may have largely focused on selecting talent later in the pipeline globally instead of investing in talent early in US K-12 education because much of the talent they are interested in (and meets company needs) comes from countries outside the US. For example, 37% of the US Nobel Prize winners from 2000–2020 in physics, chemistry, and medicine were immigrants (National Foundation for American Policy 2020). In 2016–2017, foreign students accounted for 54% of master’s degrees and 44% of doctorate degrees given in STEM fields in the US (Congressional Research Service 2019), and many top companies are founded by immigrants (Wadhwa et al. 2007). Not only do these highly gifted immigrants who are educated in the K-12 systems of other countries contribute disproportionately to US innovation; they also often end up residing in the US and having children, and many of those children are highly talented individuals who may also contribute to further innovation, what Anderson (2004, p. 15) has called the multiplier effect. Historically the US has been a magnet for highly skilled individuals in search of opportunity and who have sought out US higher education, which is still among the best in the world. However, in the broader interest of solving worldwide problems there is no reason why the US will be where individuals seek to further their personal opportunities. For solving global real-world problems, the key is that top talent is provided support to innovate wherever they are or wish to live and work.

## 7. Lack of Development of the Gifted, Particularly among the Disadvantaged

Underdevelopment of talent is a larger problem in countries outside the US—specifically low-income, low-opportunity countries (Rosling et al. 2018). However, both the students themselves and the country or world as a whole still can benefit from investing in the relatively disadvantaged talented students within the country. This should be done not just for innovation purposes, but for the purposes of equity and seeking to ensure social mobility and that positions of leadership in US society can be accessed by talented students from low-income backgrounds and other marginalized communities, especially underrepresented minorities. Here we discuss the US as we are most familiar with it, but structural and systemic barriers to talent development globally are equally important to consider.

The federal K-12 investment in gifted and talented education in the US has remained at roughly 0.0002% for decades, which amounts to 1 dollar for every $500,000 spent (Wai and Worrell 2016). This lack of investment in gifted education primarily impacts public school gifted programming, which is what most talented students from poor backgrounds rely on (Peters 2021). At the same time, talented students with parents with greater resources have not been set back by this lack of funding since their parents can find ways to provide a sufficient educational dosage for them outside traditional public schools (Berner 2017). Early universal screening for gifted and talented students coupled with adequate matching of educational programming would do a great deal to help talented-but-disadvantaged students develop to their fullest and improve the likelihood they can ascend the highly competitive elite college admissions hurdles and find their way into positions of leadership in US society. At present, however, many talented-but-disadvantaged students still fall through the cracks.

The issue of *how* and *why* gifted students from some groups are less likely to be identified is complex and controversial (see e.g., Hair et al. 2015; Liu and Waller 2018, for discussion). Societal and structural inequalities including poverty lead to gaps in identification through many mechanisms and hurdles throughout the path to being identified as gifted, but the mechanism relating most to cognitive aptitudes (and thus most relevant to this article) is clear: students from disadvantaged backgrounds are less likely to undergo cognitive testing for potential gifted identification in the first place (Card and Giuliano 2016; Grissom and Redding 2016; McBee et al. 2016). For instance, as Worrell and Dixson (2018) noted, academic achievement gaps between ethnic groups in US schools are large, and given that early educational performance (e.g., grades) is often used as evidence to nominate a child for gifted evaluation, many Black and Hispanic students are less likely to ever even be given aptitude tests. At times, families play a strong role in nomination for gifted evaluation as well, and students from low-socioeconomic status (SES) homes are less likely to have parents who push for such evaluation (Calarco 2018; Grissom and Redding 2016; McBee et al. 2016). This latter mechanism may also explain why the test used for admission into New York City’s selective high schools—schools known to have few Black and Hispanic students (e.g., Shapiro 2019)—is only taken by a relatively small proportion of students from those ethnic groups to begin with.

Once a student is evaluated for giftedness, the identification criteria vary widely. Traditional cognitive tests are likely to leave out an important population of gifted students. Almost all standardized tests that are used for various forms of educational selection include primarily math and verbal reasoning measures (Lakin and Wai 2020; Wai and Lakin 2020), leaving out spatial reasoning and other aptitudes. In the hierarchical model (Carroll 1993), below the general factor the three main specific aptitudes are math, verbal, and spatial in the Radex configuration (Lubinski 2004). Through this lens, Lakin and Wai (2020) estimated, based on three independent population representative samples, that over 2 million spatially talented students, who are adept at being able to visualize and rotate figures in their mind’s eye and work with their hands, are currently missed in US K-12 education. Therefore, curricula are not set up to suit their strengths, and these students tend to underachieve and are more likely to develop behavioral issues (Lakin and Wai 2020). This is despite the fact that spatial reasoning has been linked to a wide range of innovation outcomes from STEM to the visual arts (Wai et al. 2009), and has been shown to be malleable (e.g., Sorby et al. 2018; Uttal et al. 2013).

## 8. Development of the Gifted on Multiple Dimensions

Although we emphasize aptitude testing in *selection* processes for gifted programming, the programming itself should go far beyond traditional academic skills. Regarding character education, we also suggest the cultivation of specific skills and tendencies that have been a focus of recent empirical research. Two especially neglected areas for talent development are intellectual humility (Leary et al. 2017) and actively open-minded thinking (Baron 2019). Those two traits both involve awareness of common biases and limitations that accompany thinking, and a consequent tendency to seek and seriously consider alternative points of view. Such a tendency may help gifted students to understand that although they are highly intelligent, they should expect to make mistakes at times, and should adjust their intellectual confidence accordingly. Intellectual humility also helps gifted students to understand the importance of domain-specific knowledge when making judgments and decisions. This helps to guard against what the philosopher Nathan Ballantyne (2019) has called *epistemic trespassing*, where people with expertise in one domain make overly confident judgments far outside that domain. As academically high-achieving students become accomplished adults, they will typically develop an area of professional focus, and should carefully consider the expertise of those in other areas. Finally, intellectual humility and actively open-minded thinking both mitigate the effects of political or other ideological polarization. Rather than dismissing different perspectives, actively open-minded thinkers deliberately search for reasons why they might be wrong, and are less likely to fall prey to errors caused by biases in reasoning (Toplak et al. 2017). Interestingly, despite their openness, they are also less likely to believe fake news stories (Bronstein et al. 2019). They seem to have the best of both worlds, then—curious and tolerant of multiple viewpoints, but able to evaluate information critically when necessary.

Intellectual humility and actively open-minded thinking are especially important to cultivate in gifted children, given research showing a lack of relationship between cognitive aptitude and *myside bias* in thinking (e.g., Stanovich et al. 2013; Stanovich and West 2008). That is, brighter students are actually not substantially better than their peers at being fair and objective when evaluating evidence and argumentation, or distancing their judgment process from their prior opinions. Instead, high cognitive aptitude may only lead gifted students to be better able to rationalize and justify their beliefs, which would feed polarization rather than attenuate it. There are many studies giving guidance on how to cultivate open-minded thinking. These studies often use the umbrella term critical thinking but include core elements of open-minded thinking. For example, Parks (2015) reviewed the critical thinking literature with a particular focus on applying it to gifted education. There has been less empirical research on the teaching of intellectual humility, but Roberts (2015) suggested that teachers should model intellectual humility themselves, encourage students to explicitly describe how and what they have learned from others, and use literature to show students rich examples of intellectual humility as well as its opposite.

Because talented individuals do end up as leaders of society (Wai 2013) in various domains of influence and also hold a large amount of resources and power (Freeland 2012; Goodhart 2020; Sandel 2020), it is important to help them understand that they are fortunate to be talented to begin with. Although they have likely worked quite hard, they started their journey with cognitive and other resources that many of the less fortunate lacked. Individuals who have a head start in life should be taught not to exploit their influence or aptitudes to the disadvantage of others. Relatedly, they may have not developed the skills required to cope with failure—an experience that they may have rarely faced, instead being consistently at the head of the class and accustomed to success. Murray (2008, p. 132) argued that “No one among the gifted should be allowed to rise to a position of influence without knowing what it feels like to fail. The experience of internalized humiliation is a prerequisite for humility.” The gifted can benefit from humility and wisdom. Perhaps one key to help talented students fail deliberately is entirely consonant with ensuring all students are fully if not more than sufficiently challenged and meeting their upper cognitive limits in schools through rigorous educational opportunities (Assouline et al. 2015; Wai et al. 2010). Another might be to help the talented but disadvantaged rise to positions of influence as they will have very likely internalized failure more readily in overcoming adversity. Failure may also be crucial to withstand, perhaps even collectively over time, in order to ultimately make a true scientific or other advance. For example, Harris (2021) explains that repeated unsuccessful efforts to develop an HIV vaccine was in fact a core catalyst for developing the scientific know-how that has led to the development of a sequence of other vaccines that led to successfully combating COVID-19.

To further address polarization, gifted students—like all students—should be educated to value and respect different ways of thinking. In particular, it is important for the gifted 5 percent of achievers to have compassion for those who are not as gifted and who likely face many more challenges throughout their lives because they do not have this cognitive or other head start. The gifted should recognize that though they have earned some of their station in life, being a good citizen may increase their responsibility to care for the common good, given that they started on second or third base. This may lead to solving consequential real-world problems that can improve the common good.

## 9. Practical Implications

### 9.1. Identification of Gifted Students (and Really All Students) on a Developmental Continuum

First, students with high potential must be accurately identified. Research has repeatedly shown that formal assessments capture students that are missed through teacher nomination processes, and formal assessments also lead to more equitable identification rates across ethnic groups (e.g., Card and Giuliano 2016; Grissom and Redding 2016; McBee et al. 2016). Schools should therefore be universally screening students for high aptitude (Card and Giuliano 2016; Dynarski 2018), and also comparing students to others with similar opportunities to learn using local norms to further broaden the group of those identified and are ready for more challenging educational opportunities (Peters et al. 2019). Screening all students at an early age, on mathematical, verbal, and spatial reasoning, and then matching those students to the right mix or dosage of appropriate learning opportunities, can do a great deal to help develop their talents to the fullest (Wai and Lakin 2020). Testing at more than one point in time is important as well, to make room for late bloomers and to ensure educational programming is matched to short-term developmental need (Kaufman 2013). More generally, individuality is wide ranging and society should encourage multiple forms of talent and find productive ways to encourage intellectual diversity. This screening and support should apply to all students in schools, not just a somewhat arbitrarily defined set of students. As Sternberg (2020) noted, real-world problems often have features that are not found in typical intellectual and academic test items, and so we should always be open to considering new aptitude-related constructs and measures that can supplement current testing.

Assessing multiple areas of aptitude (even just the primary three mentioned—mathematical, verbal, and spatial) also helps to address concerns that gifted students who have concomitant disabilities (“twice-exceptional” students) are being neglected. For instance, if only one measure of aptitude is used, and it is heavily verbally loaded, a gifted student with autism spectrum disorder may not be properly identified (see Dawson et al. 2007). This does not mean that the standards for giftedness or disability identification should vary from student to student (see Lovett 2013, for some of the problems with such approaches), only that when selecting assessment measures, different areas of aptitude and disability should be considered.

### 9.2. The Imperative of Gifted Support

Second, formal gifted education should be available in far more school districts; it should be a very rare school where a student cannot access some type of appropriate talent development. Additionally, programming for supporting gifted students comes in a variety of forms and may not be limited to public schools (Berner 2017). For instance, acceleration involves leading high-aptitude learners through academic material at faster rates than their peers (Assouline et al. 2015); this broad class of interventions has relatively clear benefits for academic skill development without negative socioemotional effects (Bernstein et al. 2020; Steenbergen-Hu and Moon 2011). Enrichment strategies instead provide additional information on topics covered in class, exposing academically gifted students to specific content domains of knowledge in greater depth; this intervention is associated with even greater gains in academic skills, as well as improved socioemotional development (Kim 2016). Both strategies address the needs of the academically achieving 5 percent, replacing potentially redundant content with more challenging and stimulating work. Enrichment programs can also involve introducing high-aptitude learners to real-world problems that they may later choose to investigate in greater depth. In addition, both enrichment and acceleration can expose gifted students to quite difficult material, teaching the coping skills and self-awareness that come with the experience of making mistakes and struggling with conceptual complexity, and ultimately learning to fail productively.

### 9.3. An Environment Supporting Significant Intellectual Accomplishment

Finally, there needs to be a valuing and respect and even celebration for high accomplishments in cognitive and academic domains of expertise. Optimally, this would happen in the larger culture, but at the very least, schools should be settings where high-aptitude students are motivated to achieve appropriately ambitious goals through incentives, including attention, recognition, and praise from educational professionals and their peers. Gagné (2018) emphasized the importance of personal excellence goals in talent development, but without some extrinsic reinforcers, gifted students are apt to fall into the common path of underachievement (Siegle 2018).

## 10. Conclusions

Improving the talent development of the top 5 percent of gifted students globally will improve the likelihood of solving multiple (including presently unforeseen) consequential real-world problems in the future that can promote the common good and enhance our standard of living. Fully developing the talent of low-income and disadvantaged students is crucially important for equity reasons such as social mobility and will also improve innovation, injecting more diverse talent that has likely overcome more failures and developed character in positions of leadership. Investing in all individuals can also have numerous, broad beneficial spillover effects such as social returns. Finally, apart from the benefit to society of fully developed gifted students, the realization of one’s personal and intellectual capacities is important to support for *all* students, and for this reason alone we should be ensuring we help the most brilliant students from every walk of life have the opportunity to become their very best.

## Data Availability

Not applicable.
